# Reply to Treasure et al. Comment on “Ambrogi et al. Lung Metastasectomy: Where Do We Stand? Results from an Italian Multicentric Prospective Database. *J. Clin. Med.* 2024, *13*, 3106”

**DOI:** 10.3390/jcm13237183

**Published:** 2024-11-27

**Authors:** Marcello Carlo Ambrogi, Vittorio Aprile, Stefano Sanna, Sergio Nicola Forti Parri, Giovanna Rizzardi, Olivia Fanucchi, Leonardo Valentini, Alberto Italiani, Riccardo Morganti, Carlotta Francesca Cartia, James M. Hughes, Marco Lucchi, Andrea Droghetti

**Affiliations:** 1 Department for Surgical, Medical, Molecular Pathology and Critical Care, University of Pisa, 56124 Pisa, Italy; 2 Division of Thoracic Surgery, University Hospital of Pisa, 56124 Pisa, Italy; 3 Multispecialistic Surgical Department, Private Forlì Hospitals, 47122 Forlì, Italy; 4 Department of Thoracic Surgery, IRCCS University Hospital of Bologna, 40138 Bologna, Italy; 5 Division of Thoracic Surgery, Humanitas Gavazzeni Hospital, 24125 Bergamo, Italy; 6 Statistical Support Division for Clinical Studies, University Hospital of Pisa, 56124 Pisa, Italy; 7 Division of Thoracic Surgery, Candiolo Cancer Institute, FPO-IRCCS, 10060 Candiolo, Italy

We would like to express our sincere gratitude for the thoughtful reflections on our recent study regarding pulmonary metastasectomy, and we greatly appreciate the constructive dialog that our work has sparked.

That being said, we believe the author’s commentary has taken a somewhat one-sided approach, focusing on a single aspect of our study rather than acknowledging the broader context of our work, which has involved over a decade of research and collaboration with numerous investigators [1].

Our aim in this response is, first and foremost, to address the specific points raised by the author. Additionally, we believe it is important to clarify the intent behind certain comments, which may have been misinterpreted.

To begin with, there was certainly no intention to show disrespect toward the commenter when citing the results of the PulMiCC study [2]. We acknowledge the value of the study and recognize its significant contribution to the ongoing discourse on the role of surgery in the treatment of pulmonary metastases, particularly those of colorectal origin. The randomized controlled trial was a substantial effort, and we fully appreciate the challenges the authors faced in its execution [3].

However, while PulMiCC has provided critical insights, it is important to note that it is not the only study addressing this topic. Several other investigations, involving larger patient cohorts, have demonstrated different outcomes, many of which are consistent with our own findings [4,5,6]. It is therefore legitimate to raise the doubt as to whether a single randomized controlled trial can surpass and refute years of extensive, multicentric, and reliable retrospective studies.

In our recent article, we simply reported the conclusions from the PulMiCC trial, specifically noting: “Because of poor and worsening recruitment, the study was stopped. The small number of participants in the trial precludes a conclusive answer to the research question given the large overlap in the confidence intervals in the proportions still alive at all time points”.

Furthermore, we did not mean to disparage the author’s efforts by citing this study without referencing others that followed. Indeed, even in later publications, various researchers have questioned both the role of metastasectomy and the broader impact of the PulMiCC study on daily clinical practice [7,8].

While we fully support evidence-based medicine, we do not believe that a single study should lead to the dismissal of surgical interventions for pulmonary metastases. To this day, we continue to encounter cases—especially in colorectal cancer—where surgery remains a viable and, in some instances, the best therapeutic option. As Gray and colleagues have noted, despite the debates, surgery still represents a potential path to cure in selected patients [9].

Our study aimed to emphasize that in carefully selected patient groups, surgery continues to play a meaningful therapeutic role. We believe that any scientific discussion should be balanced, incorporating diverse perspectives to provide a comprehensive view of the subject. At the same time, we must be mindful of editorial constraints, which can limit the length of articles and the number of references.

Lastly, we were somewhat surprised to see our study referenced by the author in another article as an example of a paper published in what was described as a “predatory” journal [10]. Without delving into the broader debate about such journals—an issue on which we likely share some of the same concerns—it is essential to maintain a respectful dialog. Every article, regardless of where it is published, deserves to be assessed on its merits, and we trust in the readers’ ability to critically evaluate the content of any paper.

Thank you once again for your engagement with our work and for contributing to this important debate.

## Data Availability

Data supporting the findings of this study are available from the authors on reasonable request.
